# Prevalence and Clinical Predictors of Psoriatic Arthritis in Saudi Patients With Psoriasis: A Single-Center Retrospective Cohort Study

**DOI:** 10.7759/cureus.46632

**Published:** 2023-10-07

**Authors:** Mishari T Alrubaiaan, Saad A Alsulaiman, Abdullah Alqahtani, Abdullah N Altasan, Faisal O Almehrij, Abdulrahman Alrashid, Osama L Mohamed

**Affiliations:** 1 College of Medicine, King Saud Bin Abdulaziz University for Health Sciences, Riyadh, SAU; 2 Rheumatology, King Abdulaziz Medical City, Riyadh, SAU; 3 Biostatistics, King Abdullah Biography Center, Riyadh, SAU

**Keywords:** clinical predictors, pso, psa, psoriatic arthritis, psoriasis, prevalence

## Abstract

Background

Among psoriatic patients, psoriatic arthritis is the most common and most impactful comorbidity. In most cases, it occurs after the onset of psoriasis. Detecting and treating it early is crucial for rheumatologists and dermatologists.

Objectives

The study aimed to determine the prevalence of psoriatic arthritis among recognized cases of psoriasis, as well as to determine the clinical features of psoriasis that are linked to a greater prevalence of psoriatic arthritis at King Abdulaziz Medical City (KAMC), Riyadh.

Methods

A retrospective cohort study of 487 psoriatic patients diagnosed between 2015 and 2023 was conducted at KAMC, Riyadh. The study included subjects aged 18 years or older with a psoriasis diagnosis documented by a dermatologist and a psoriatic arthritis diagnosis documented by a rheumatologist based on the Classification Criteria for Psoriatic Arthritis (CASPAR). Patients younger than 18 years, diagnosed with psoriatic arthritis concurrently with psoriasis, or within 90 days of psoriasis diagnosis, or who lack a documented diagnosis of psoriasis by a dermatologist were excluded. The study evaluated demographic data and medical variables concerning psoriasis (age at onset, type of psoriasis, site of psoriasis, and nail dystrophy) and psoriatic arthritis. SPSS Statistics version 25 (IBM Corp., Armonk, NY) was used to conduct statistical analysis. The p-value of 0.05 was used to evaluate statistical significance.

Results

Overall, 487 patients had psoriasis in this study. Of these, 49 (10%) were diagnosed with psoriatic arthritis. The mean ± standard deviation of the age of the psoriasis group was 41.7 ± 15.6 years, with 264 (54.2%) females and 223 (44.8%) males. The clinical features of psoriasis that were linked to a greater frequency of psoriatic arthritis in our study included female gender (71.4%), plaque psoriasis (95.9%), psoriatic lesions involving the extremities (75%), scalp (42.9%), and trunk (36.7%), nail dystrophy (28.6%), as well as the involvement of three or more sites (40.8%) at the time of their initial diagnosis of psoriasis.

Conclusion

Our study indicated that 10% of Saudi patients with psoriasis had psoriatic arthritis. Moreover, the present study shows that patients with greater psoriatic lesions at initial presentation are more likely to develop psoriatic arthritis.

## Introduction

A chronic inflammatory skin disease, known as psoriasis, has a strong genetic predisposition and autoimmune pathogenic characteristics [1]. The prevalence of psoriasis fluctuates from one country to another; however, it is approximately 2% [2]. Psoriasis can also affect various demographic groups to different extents [2]. In Saudi Arabia, psoriasis was found to affect approximately 5.33% in the northern region, and 3.4% and 1.5% in the southern and eastern regions, respectively [2].

Typically, joint involvement associated with psoriatic lesions is referred to as psoriatic arthritis [3]. Psoriatic arthritis was first studied in France by Bourdillon et al. (1994) [4]. Initially, it was regarded as one of the manifestations of rheumatoid arthritis [4]. However, in recent years, psoriatic arthritis has been considered a separate disease affecting around 2-26% of individuals with psoriasis [5,6]. In addition, there are reports of higher rates of psoriasis and joint manifestations consistent with psoriatic arthritis in other studies, as much as 48% [5,6]. Arthritis usually tends to develop after the occurrence of psoriasis in a couple of years [3,4]. However, in a small portion of psoriatic patients, it occurs at the same time as psoriasis [3,4].

Psoriatic arthritis risk factors, development, and progression have been investigated in several studies [7]. Conversely, studies focusing on the clinical features of psoriasis that are linked to a greater frequency of psoriatic arthritis are limited [7]. According to a study conducted by Wilson et al. (2009), patients with nail involvement, scalp lesions, and perianal psoriasis showed a higher frequency of developing psoriatic arthritis [7]. The involvement of the nails in psoriatic arthritis varies across different studies. Pitting was found to be the most frequent followed by onycholysis and leukonychia [8,9]. Despite the lack of clarity regarding the reasons behind the potential association between nail involvement and an increased risk of psoriatic arthritis, the condition may operate as a sign of elevated immunoreactivity in psoriasis [7]. Furthermore, patients with severe psoriasis are more likely to develop psoriatic arthritis than those with mild psoriasis [7,9]. Moreover, if oral corticosteroid use occurs within two years of diagnosis, the risk of psoriatic arthritis increases [7]. Pregnancy, however, is negatively associated with psoriatic arthritis onset [7,9-11]. More recently, psoriatic arthritis has been linked to acute stressors and physical trauma in patients with psoriasis [7,9,11].

Our study aimed to determine the overall frequency of psoriatic arthritis among documented cases of psoriasis, as well as to identify the clinical features of psoriasis that are linked to a greater frequency of psoriatic arthritis at King Abdulaziz Medical City (KAMC), Riyadh.

## Materials and methods

Study design

This retrospective cohort study included subjects with psoriasis diagnosis documented in the BESTCare system from 2015 to 2023 at KAMC, Riyadh. Using the BESTCare system, all documented cases of psoriasis and psoriatic arthritis can be captured, with full access to medical records.

Selection criteria

The study consisted of patients aged 18 years or older with a psoriasis diagnosis documented by a dermatologist and a psoriatic arthritis diagnosis confirmed by a rheumatologist based on the Classification Criteria for Psoriatic Arthritis (CASPAR). In the study, patients younger than 18 years, patients diagnosed with psoriatic arthritis concurrently with psoriasis, or within 90 days of psoriasis diagnosis, or patients who lack a documented diagnosis of psoriasis by a dermatologist were excluded.

Sample size

There are 867 cases of psoriasis at KAMC. So, the required sample size was estimated using the Raosoft sample size calculator (Raosoft, Inc., Seattle, WA). It was determined that at least 355 patients with psoriasis were required, and after meeting the inclusion and exclusion criteria, our sample size was 487 patients with a psoriasis diagnosis.

Data collection and analysis

The data of all patients with psoriasis diagnosis was obtained by the study group from the BESTCare system after getting IRB approval. A data abstraction form was utilized to collect demographic information, clinical manifestations, psoriasis type, location, and involvement of nails [7]. The type of psoriasis was determined by the physician's diagnosis or description of lesions in the medical records. It was further divided into chronic plaque psoriasis, guttate psoriasis, sebopsoriasis, pustular psoriasis, erythrodermic psoriasis, and inverse psoriasis [7]. Chronic plaque psoriasis was assumed when medical records failed to specify the type of psoriasis [7]. In addition, we reviewed the medical records of patients with psoriasis and psoriatic arthritis. Only patients who had a confirmed diagnosis of psoriatic arthritis by a rheumatologist and who fulfilled the CASPAR criteria were enrolled. Among the data collected were demographics, clinical characteristics of psoriatic arthritis such as joint pattern and dactylitis, lab results such as rheumatoid factor (RF), and radiographic characteristics of psoriatic arthritis [7].

Statistical analysis

Descriptive statistics such as mean, median, and standard deviations were used to summarize the data. Variations between groups were examined using the chi-square test and Fisher's exact test. The threshold for statistical significance was set at 5% and all tests were two-tailed. For all statistical analyses, SPSS Statistics version 25 (IBM Corp., Armonk, NY) was used.

Ethical consideration

Ethical and Institutional Review Board (IRB) approval was granted by the King Abdullah International Medical Research Center (reference number: NRC23R/308/05).

## Results

The study population consisted of 487 psoriasis patients first diagnosed between 2015 and 2023. In this cohort, we identified 49 subjects with a rheumatologist-confirmed diagnosis of psoriatic arthritis and validated their diagnosis using the CASPAR criteria.

Table 1 describes the clinical characteristics of 487 subjects upon diagnosis of psoriasis and without the presence of psoriatic arthritis. The mean ± standard deviation of the age of the psoriasis cohort was 41.7 ± 15.6 years, and 264 (54.2%) were female while 223 (45.8%) were male. A total of 427 (87.7%) of the subjects were diagnosed with plaque psoriasis, followed by 32 (6.6%) with guttate psoriasis, nine (1.8%) with pustular psoriasis, eight (1.6%) with inverse psoriasis, five (1%) with erythrodermic psoriasis, and three (0.6%) with sebopsoriasis. At initial presentation, the most prevalent location of psoriasis was the extremities in 304 (62.4%), followed by the scalp in 175 (35.9%), the trunk in 150 (30.8%), palms and/or soles in 61 (12.5%), face in 46 (9.4%), intergluteal and/or perianal in 30 (6.2%), and axilla and/or groin in 18 (3.7%) patients. A total of 187 (38.1%) patients presented with three or more affected sites at the initial presentation. Of the subjects, 98 (20.1%) presented with nail dystrophy at the initial presentation.

**Table 1:** The clinical characteristics of 487 subjects with psoriasis and without psoriatic arthritis at the time of psoriasis diagnosis. Values are represented as numbers (percentages) unless otherwise indicated. Age is represented as mean and standard deviation (SD).

Total, N (%)	487 (100)
Age, years	
Mean (SD)	41.7 (15.6)
Median (minimum, maximum)	40 (14, 95)
Age category, N (%)	
<40	232 (47.6)
≥40	255 (52.4)
Gender, N (%)	
Male	223 (45.8)
Female	264 (54.2)
Type of psoriasis, N (%)	
Plaque	427 (87.7)
Guttate	32 (6.6)
Sebopsoriasis	3 (0.6)
Pustular	9 (1.8)
Erythrodermic	5 (1)
Inverse	8 (1.6)
Site of psoriasis, N (%)	
Scalp	175 (35.9)
Extremities	304 (62.4)
Trunk	150 (30.8)
Intergluteal/perianal	30 (6.2)
Face	46 (9.4)
Palms and/or soles	61 (12.5)
Axilla/groin	18 (3.7)
Unknown	32 (6.6)
Nail dystrophy, N (%)	98 (20.1)
Number of affected sites, N (%)	
1 site	120 (24.6)
2 sites	136 (27.9)
≥3 sites	187 (38.4)
Unknown	44 (9)

Table 2 outlines the clinical features of psoriasis that are correlated with a higher frequency of psoriatic arthritis in psoriatic patients. The female gender was found to have a higher prevalence of psoriatic arthritis (35, 71.4%). Further, 47 (95.9%) of the subjects with psoriatic arthritis had plaque psoriasis while only two (4.1%) had guttate psoriasis. Initial psoriatic locations that were correlated with a greater frequency of psoriatic arthritis were the extremities in 37 (75.5%), followed by the scalp in 21 (42.9%) subjects, and the trunk in 18 (36.7%) subjects. Only three (6.1%) subjects with psoriatic arthritis had intergluteal and/or perianal psoriatic lesions at the initial presentation. Psoriatic arthritis was more prevalent in psoriasis patients with three or more affected sites at initial presentation (20, 40.8%). Additionally, 14 (28.1%) patients with psoriatic arthritis had nail dystrophy at initial presentation.

**Table 2:** The clinical characteristics associated with a higher prevalence of psoriatic arthritis in the psoriatic group. Values are represented as numbers (percentages) unless otherwise indicated. Age is represented as mean and standard deviation (SD).

Confirmed diagnosis of psoriatic arthritis by a rheumatologist	Yes	No
Total, N (%)	49 (10.1)	438 (89.9)
Age, mean (SD)	42.8 (14.4)	41.6 (15.8)
Age category, N (%)		
<40	19 (38.8)	213 (48.6)
≥40	30 (61.2)	225 (51.4)
Gender, N (%)		
Male	14 (28.6)	209 (47.7)
Female	35 (71.4)	229 (52.3)
Type of psoriasis, N (%)		
Plaque	47 (95.9)	380 (86.8)
Guttate	2 (4.1)	30 (6.8)
Sebopsoriasis	0 (0)	3 (0.7)
Pustular	0 (0)	9 (2.1)
Erythrodermic	0 (0)	5 (1.1)
Inverse	0 (0)	8 (1.8)
Site of psoriasis, N (%)		
Scalp	21 (42.9)	154 (35.2)
Extremities	37 (75.5)	267 (61)
Trunk	18 (36.7)	132 (30.1)
Intergluteal and/or perianal	3 (6.1)	27 (6.2)
Face	2 (4.1)	44 (10)
Palms and/or soles	8 (16.3)	53 (12.1)
Axilla and/or groin	3 (6.1)	15 (3.4)
Unknown	2 (4.1)	30 (6.8)
Nail dystrophy, N (%)	14 (28.6)	84 (19.2)
Number of affected sites, N (%)		
1 site	9 (18.4)	111 (25.3)
2 sites	13 (26.5)	123 (28.1)
≥3 sites	20 (40.8)	167 (38.1)
Unknown	7 (14.2)	37 (8.4)

In Table 3, we compared the clinical and radiological findings of 30 patients who were diagnosed with psoriatic arthritis within one year of psoriasis onset versus 19 patients who were diagnosed with psoriatic arthritis after more than one year of psoriasis onset. Subjects who were diagnosed with psoriatic arthritis within one year of psoriasis onset were younger (42.87 vs. 45.26, P = 0.574), more likely to be female (73.3% vs. 68.4%, P = 0.711), and more likely to complain of central arthritis (53.3% vs. 42.1%, P = 0.561) and dactylitis (63.3% vs. 47.4%, P = 0.376). Moreover, in total, 24 patients with a confirmed diagnosis of psoriatic arthritis (16 of whom had confirmed sacroiliitis through imaging) had central arthritis (P = 0.444), whereas 28 patients had peripheral arthritis (P = 0.612). Furthermore, 28 patients had dactylitis (P = 0.271) and five had enthesitis (P = 1).

**Table 3:** Clinical characteristics of psoriatic arthritis (PsA) within psoriasis subjects. Values are represented as numbers (percentages) unless otherwise indicated. Age is represented as mean and standard deviation (SD). * Fisher’s exact test was used for the p-value. A p-value less than 0.05 was considered statistically significant.

Variable	PsA within one year of psoriasis onset (n = 30)	PsA after more than one year of psoriasis onset (n = 19)	p-value
Age at diagnosis of psoriasis, mean (SD)	42.63 (14.7)	42.95 (14.2)	0.941
Age at diagnosis of psoriatic arthritis, mean (SD)	42.87 (14.7)	45.26 (14.0)	0.574
Gender, N (%)			
Male	8 (26.7)	6 (31.6)	0.711
Female	22 (73.3)	13 (68.4)	0.711
Age category, N (%)			
<40	12 (40.0)	6 (31.6)	0.551
>40	18 (60.0)	13 (68.4)	0.551
Central arthritis, N (%)	16 (53.3)	8 (42.1)	0.444
Peripheral arthritis, N (%)	12 (40.0)	9 (47.4)	0.612
Enthesitis, N (%)	3 (10.0)	2 (10.5)	1*
Dactylitis, N (%)	19 (63.3)	9 (47.4)	0.271
Positive rheumatoid factor, N (%)	0 (0.0)	1 (6.3)	0.39*
Nail dystrophy, N (%)	8 (26.7)	3 (15.8)	0.492*
Radiologic findings, N (%)	11 (36.7)	7 (36.8)	0.99
Radiologic erosions, N (%)	5 (16.7)	2 (10.5)	0.691*

## Discussion

In this research, we undertook an examination of the overall prevalence of psoriatic arthritis among documented cases of psoriasis. As well, we determined the distinctive clinical features of psoriasis that were linked to a greater frequency of psoriatic arthritis. It was observed that approximately 10% of individuals diagnosed with psoriasis eventually developed psoriatic arthritis over the course of their lives. Factors such as female gender and the presence of plaque psoriasis had a greater frequency of developing psoriatic arthritis. Moreover, in our study, we found that psoriatic lesions that involved the extremities at initial presentation had the highest frequency of developing psoriatic arthritis, followed by psoriatic lesions that involved the scalp and trunk, respectively. Additionally, individuals who exhibited three or more affected sites at the onset of their psoriasis condition, along with those experiencing nail dystrophy, displayed a notably elevated prevalence of psoriatic arthritis. These findings suggest that individuals with greater psoriasis manifestations are at an increased frequency of developing psoriatic arthritis.

It is important to gain a comprehensive understanding of the prevalence of psoriatic arthritis among patients with psoriasis when investigating the epidemiological aspects of both conditions [7]. Existing approximations of the prevalence of psoriatic arthritis among individuals with psoriasis have significant variations; however, it ranges from 7% to 40% [5-7].

Moreover, our research findings indicate that women may exhibit a slightly greater predisposition to develop psoriatic arthritis in comparison to men. Notably, a limited number of research have ventured into exploring the role of hormonal factors in psoriasis [10,12,13]. A study encompassing a cohort of 33 females with psoriatic arthritis revealed that they were more likely to have disease flares when estrogen levels were at their lowest [13]. Furthermore, in a study conducted by Ostensen (1988), he reported that most of his patients experienced clinical improvements in psoriatic arthritis during pregnancy [13]. Similarly, Thumboo et al. (2002) reported that women who got pregnant while having psoriasis were less likely to have psoriatic arthritis [10]. These research outcomes indicate a potential role of elevated estrogen levels in mitigating the occurrence of psoriatic arthritis [7]. Nevertheless, it is imperative to note that the epidemiological observations available to date remain insufficient in terms of elucidating the precise impacts of estrogen and other sex hormones [7].

Numerous investigations have been conducted to explore the risk factors associated with the onset and advancement of psoriatic arthritis [7]. Nonetheless, limited studies have explored the clinical features of psoriasis that were linked to a higher prevalence of psoriatic arthritis [7]. In our study, we observed a higher prevalence of psoriatic arthritis among patients who were female (71.4%), exhibited plaque psoriasis (95.9%), presented with psoriatic lesions affecting the extremities (75%), scalp (42.9%), and trunk (36.7%), reported nail dystrophy (28.6%), or had involvement of three or more anatomical sites (40.8%) at the initial manifestation of psoriasis. A study by Wilson et al. (2009) involved 1593 subjects with psoriasis, of which 97 were confirmed to have psoriatic arthritis [7]. This study showed a significant association between scalp lesions, intergluteal and/or perianal lesions, nail dystrophy, and the involvement of three or more anatomical sites and psoriatic arthritis [7]. Our findings align with prior research, which has consistently shown that larger and more severe psoriatic lesions correlate with an increased risk of psoriatic arthritis [6,7,14,15]. The presence of larger psoriatic lesions may be implicated in the elevation of tumor necrosis factor-alpha (TNF-α) levels, which have been observed in the synovium, synovial lining, and skin lesions of individuals diagnosed with psoriatic arthritis [7,16,17]. Consequently, these elevated TNF-α levels may contribute to the pathogenesis of psoriatic arthritis [7,16,17].

Moreover, it is noteworthy to indicate that in our investigation, only 28.6% of subjects diagnosed with psoriatic arthritis reported nail involvement as their initial manifestation of psoriasis. A comprehensive long-term study identified an association between the development of psoriatic arthritis and the presence of nail psoriasis [7]. Wilson et al. (2009) originally reported the predictive value of nail psoriasis in the onset of psoriatic arthritis [7]. Among the various nail abnormalities documented in previous research, nail pitting emerged as the most prevalent, followed by onycholysis as the next commonly observed nail abnormality [8]. Additionally, nail symptoms such as leukonychia (28.42%), oil drop patterns (21.29%), and splinter hemorrhages (18.36%) exhibited consistent associations with psoriatic arthritis [9]. Although the precise link between nail dystrophy and an increased risk of psoriatic arthritis remains vague, it is plausible that this condition may serve as an indicator of heightened immunoreactivity in patients with psoriasis, ultimately predisposing them to psoriatic arthritis [7].

There are several limitations in this study. It is possible that we have missed some cases of unrecognized psoriatic arthritis since it can manifest with minimal or absent skin lesions. In addition, the body surface area (BSA) percentage and the Psoriasis Area and Severity Index (PASI) score were not included in this study. Therefore, it is difficult to accurately determine whether severe psoriasis at initial presentation is linked to psoriatic arthritis. Furthermore, since it is a retrospective study, it is prone to detection and reviewer bias, as well as variations in physician's documentation. Hence, larger prospective studies are required. Lastly, our study focused on the known baseline clinical features of psoriasis and did not take into consideration the evolution of these features over time and their corresponding treatment regimens.

## Conclusions

The purpose of this retrospective cohort study conducted at KAMC was to investigate the prevalence of psoriatic arthritis among patients with psoriasis and to further investigate the clinical features of psoriasis correlated with a greater frequency of psoriatic arthritis. Overall, the results provide some tentative support that greater psoriatic lesions at initial presentation are associated with a higher frequency of developing psoriatic arthritis. However, further prospective studies with a greater sample size should be established.
